# Supplementary material for: Machine Learning Prediction of Non-Coding Variant Impact in Cell-Class-Specific Human Retinal Cis-Regulatory Elements
Source: bioRxiv. 2025 Feb 24:2025.02.22.638679. Preprint. [Version 1] doi: 10.1101/2025.02.22.638679 (PMC11888276; doi:10.1101/2025.02.22.638679)
Supplement: Supplement 1 — Figure S1 Model Accuracy as Measured by Five-Fold Cross Validation A. Precision Recall Curve by model as determined by 5-fold cross validation of training data. B. Areas under the curve for ROC and PR curves in Figure 1C and S1A. [file media-1.pdf]

A

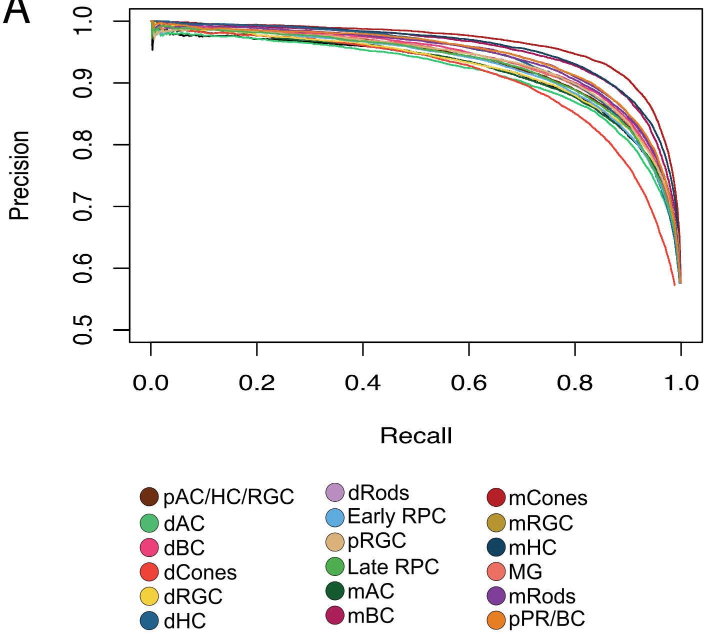

B

Cell Class	ROC AUC	PRC AUC
Early RPC	0.93	0.93
pRGC	0.94	0.94
pAC/HC/RGC	0.94	0.93
dRGC	0.93	0.92
Late RPC	0.93	0.93
pPR/BC	0.94	0.94
dHC	0.93	0.94
dAC	0.92	0.91
dCones	0.91	0.91
dRods	0.94	0.94
dBC	0.94	0.94
pRGC	0.94	0.93
mHC	0.95	0.95
mAC	0.93	0.92
mCones	0.96	0.96
mRods	0.94	0.94
MG	0.93	0.93
mBC	0.95	0.95
