# Supplementary figures and images for: Machine Learning Prediction of Non-Coding Variant Impact in Cell-Class-Specific Human Retinal Cis-Regulatory Elements
Source: bioRxiv. 2025 Feb 24:2025.02.22.638679. Preprint. [Version 1] doi: 10.1101/2025.02.22.638679 (PMC11888276; doi:10.1101/2025.02.22.638679)

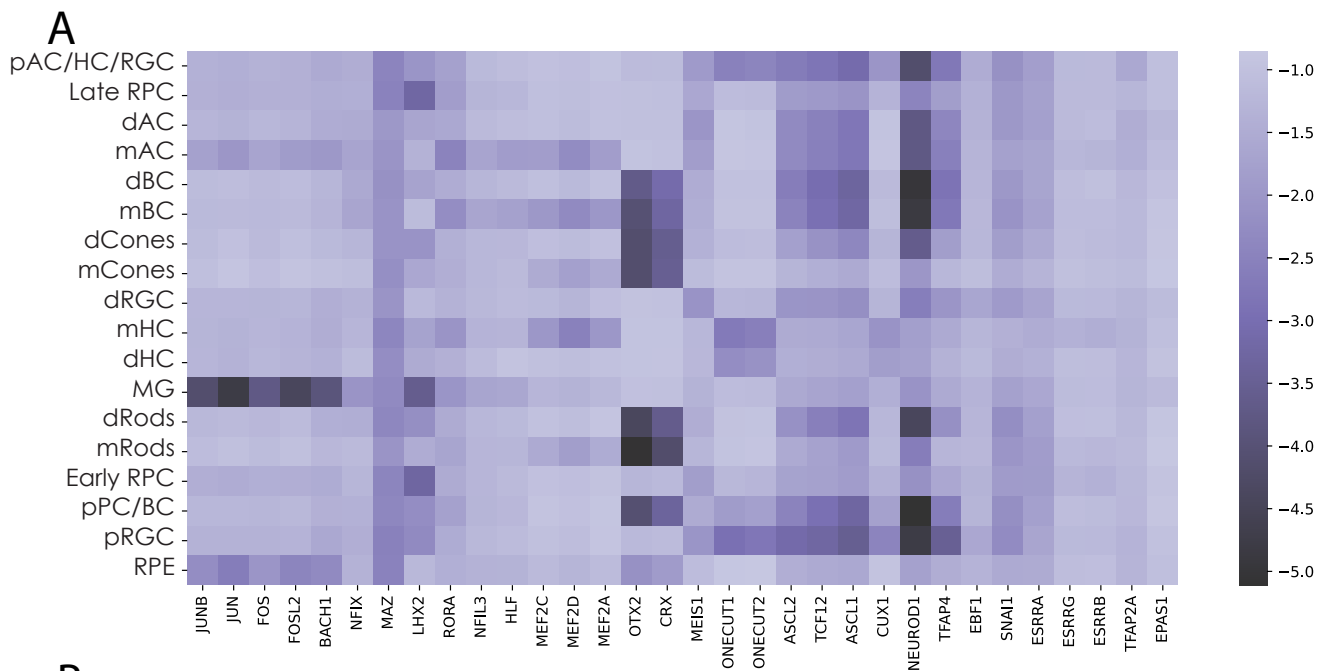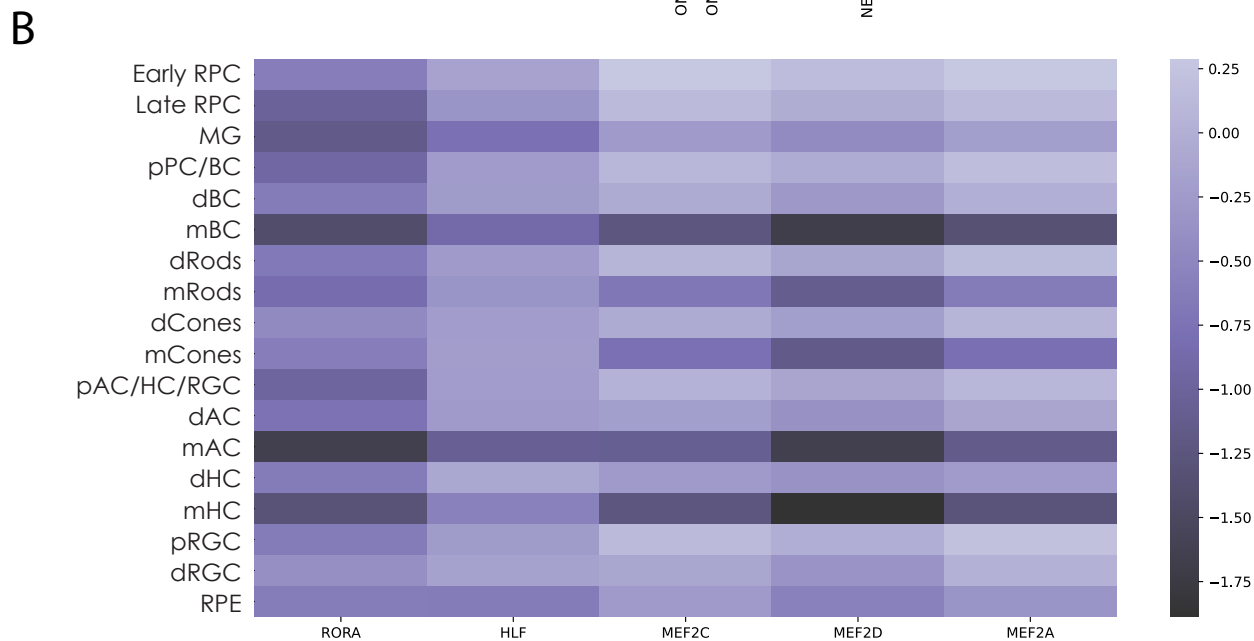

Supplement: Supplement 2 — Figure S2 Cell-class-specific Variant Impact Prediction Scoring of Critical TF Motifs A. Average VIP score for SNVs present in previously identified motifs (x-axis) as stratified by model (y-axis). B. Average VIP score for SNPs present in TF motifs for which scoring demonstrates an increased predicted impact (more negative score) in more mature cell classes as compared to developing counterparts. [file media-2.pdf]

**A**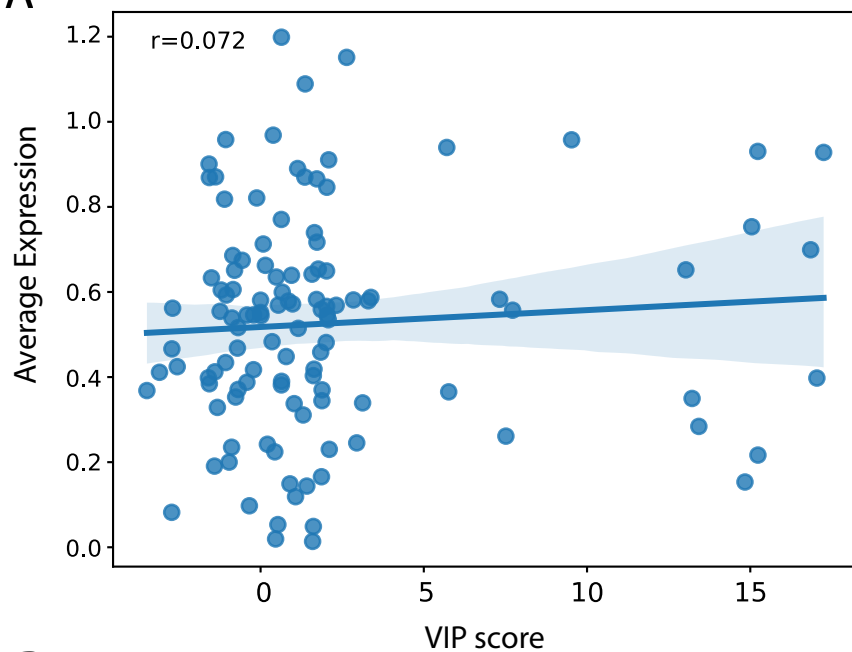**B**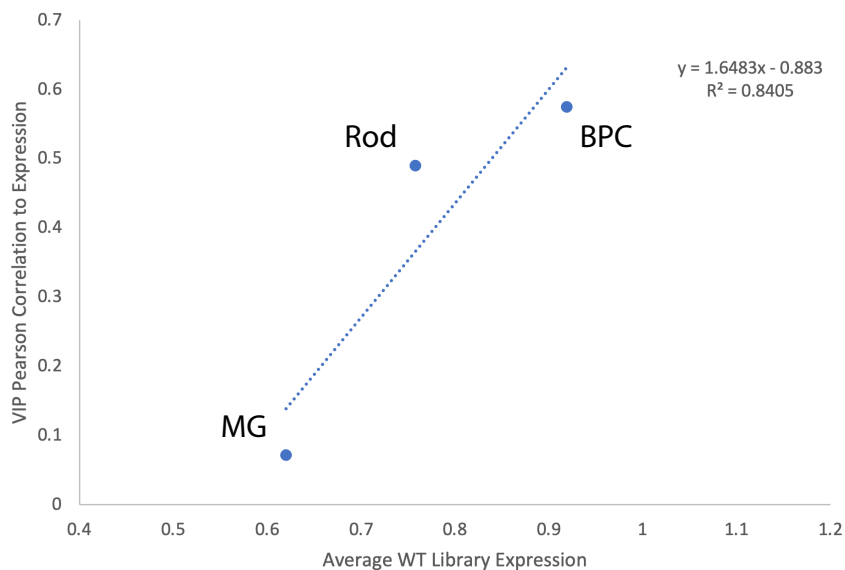

Supplement: Supplement 3 — Figure S3 Variant Impact Prediction scoring compared to massively parallel reporter assay expression by cell class. A. Scatter plot of Müller glial cell specific VIP scores at the Gnb3 promoter (x-axis) versus average variant reporter expression changes in Müller glial cells (note expression of Gnb3 variant reporters is significantly lower in Müller glial cells compared to bipolar cells and rods – see Figure 3B & C) MPRA from Zhao et al 2023 [34]. Pearson correlation in upper lefthand corner. B. Correlation of WT library expression against Pearson correlation of variant expression vs VIP. Cell types labeled by point. [file media-3.pdf]
